# HCV Innate Immune Responses

**DOI:** 10.3390/v1031073

**Published:** 2009-11-30

**Authors:** Markus H. Heim

**Affiliations:** 1 Clinic for Gastroenterology and Hepatology, University Hospital Basel, Petersgraben 4, CH-4031 Basel, Switzerland; 2 Department of Biomedicine, University Basel, ZLF, Hebelstrasse 20, CH-4031 Basel, Switzerland; E-Mail: markus.heim@unibas.ch; Tel.: +41 61 265 25 25; Fax: +41-61-265 52 53

**Keywords:** interferon, MAVS, Toll-like receptors, Jak-STAT, HCV, viral interference

## Abstract

Hepatitis C virus (HCV) establishes a persistent infection in more than 70% of infected individuals. This striking ability to evade the powerful innate immune system results from viral interference occurring at several levels of the interferon (IFN) system. There is strong evidence from cell culture experiments that HCV can inhibit the induction of IFNβ by cleaving important proteins in the virus sensory pathways of cells such as MAVS and TRIF. There is also evidence that HCV interferes with IFNα signaling through the Jak-STAT pathway, and that HCV proteins target IFN effector systems such as protein kinase R (PKR). These *in vitro* findings will have to be confirmed in clinical trials investigating the molecular mechanisms of HCV interference with the innate immune system in liver samples.

## 1. Introduction

Hepatitis C virus (HCV) infection persists in more than 70% of exposed individuals. The ability of HCV to persist within a host is striking, and is believed to be due to numerous efficient mechanisms by which the virus evades the immune response of the host. Type 1 interferons (IFNs) are crucial cytokines in innate immunity. HCV interferes with all aspects of the IFN system: with the induction of IFNβ in infected cells, with IFNα/β signaling through the Jak-STAT pathway, and with IFN induced proteins with antiviral properties.

## 2. Interferons

IFN was identified more than 50 years ago by Isaacs and Lindenmann during their studies of the phenomenon of viral interference, the ability of an active or inactivated virus to interfere with the growth of an unrelated virus [1]. Today, more than 10 mammalian IFN species and numerous subspecies have been discovered, each with individual properties, but all having antiviral activity [2]. They are currently classified into three groups: type I, type II and type III IFNs. The type I IFNs include all IFN-αs, IFN-β, IFN-ε, IFN-κ, IFN-ω and IFN-ν [3]. Humans have 12 different IFN-αs and a single IFN-β. Type I IFN genes are clustered on the human chromosome 9. Each subtype is encoded by its own gene and regulated by its own promoter, and none of them contain introns [3]. The different IFN-αs and IFN-β have substantial differences in their specific antiviral activities and in the ratios of antiviral to antiproliferative activities. However, the molecular basis of these differences is not yet known. All type I IFNs bind to the same interferon alpha/beta receptor (IFNAR) that consists of two major subunits: IFNAR1 (the a subunit in the older literature) [4] and IFNAR2c (the βL subunit) [5,6].

There is only one class II IFN, IFN-γ. IFNγ is produced by T lymphocytes when stimulated with antigens or mitogens. IFNγ binds to a distinct receptor, the interferon gamma receptor (IFNGR), that consists of the two subunits IFNGR1 (previously α chain) [7] and IFNGR2 (previously β chain or accessory factor) [8,9].

The recently described type III IFNs IFN-λ2, IFN-λ3 and IFN-λ1 are also known as IL-28A, IL-28B and IL29, respectively. Same as type I IFNs, they are also induced by viral infections [10]. They signal through the IFN-λ receptor consisting of the IL-10R2 chain that is shared with the IL-10 receptor, and a unique IFN-λ chain [11,12]. The recent discovery that a genetic polymorphism near the IL28B gene, encoding IFN-λ3, is associated with an approximately twofold change in response to treatment with pegylated IFNα/ribavirin provides strong evidence that type III IFNs are important for the control of HCV [13–15].

## 3. Induction of Type I Interferons

Cells produce IFN-αs and IFN-β in response to infection by a variety of viruses. Unlike bacteria and fungi, which have microbe-specific structures distinguishable from host cell structures, viruses are made predominantly of host-derived components. Given the lack of virus specific proteins or lipids, the cellular receptors that detect viruses have instead evolved to recognize the presence of the viral genome composed of nucleic acids. Two important pathways that detect viral genomes and induce type I IFNs have been discovered and characterized during recent years: the toll-like receptor (TLR) dependent pathway [16,17] and the cytosolic pathway triggered by binding of viral RNA to the RNA helicases retinoic acid inducible gene-I (RIG-I) and melanoma differentiation antigen 5 (MDA5) [18,19].

**Figure 1.**
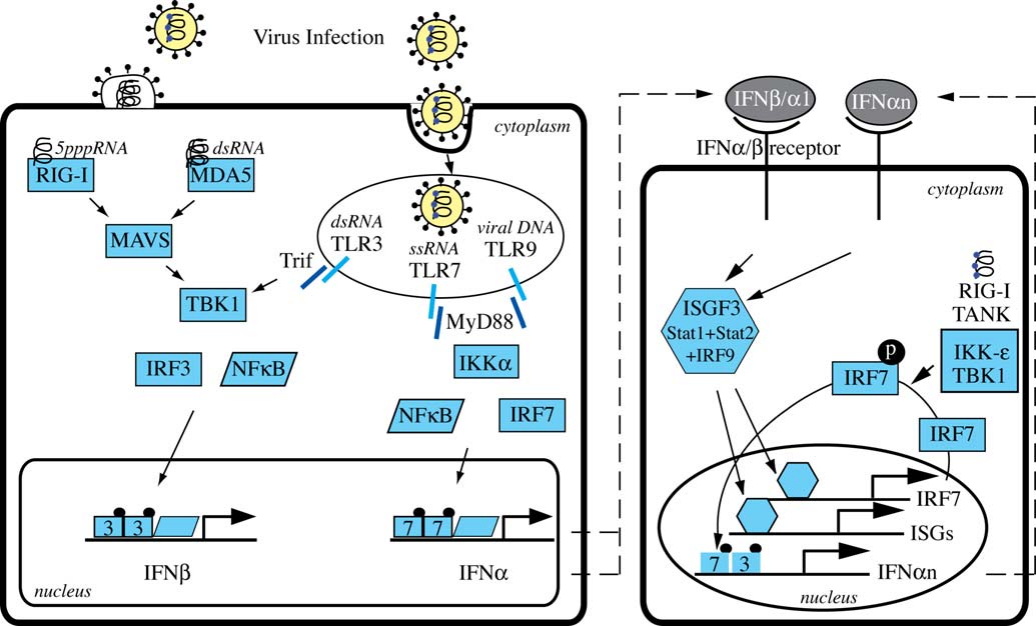
Induction of IFNβ by viral infections.

TLRs are a family of transmembrane pattern recognition receptors (PRRs) that recognize microbial pathogen associated molecular patterns (PAMPs) and activate the expression of genes involved in inflammatory and immune responses [17]. There are at least 10 human TLRs, and 3 of them are involved in the recognition of viral infections: TLR3, TLR7 and TLR9. TLRs are expressed on various immune cells such as macrophages, dendritic cells (DCs), B cells, but also on fibroblasts and epithelial cells. While TLRs involved in the recognition of bacterial components are expressed on the cell surface, TLR3, TLR7 and TLR9 are localized in intracellular compartments such as endosomes. TLR3 recognizes dsRNA (e.g. HCV-RNA)[20], TLR7 detects ssRNA [21,22] and TLR9 interacts with unmethylated DNA with CpG motifs [23]. TLR activation induces signaling cascades that mainly involve the key transcription factors NF-κB and various interferon regulatory factors (IRFs) (Figure 1). Specifically, IRF3 and IRF7 have both distinct and essential roles for virus induced transcriptional activation of IFN-β [24]. IRF3 is constitutively expressed in most cells, whereas IRF7 is expressed at low amounts and is strongly expressed only after stimulation of cells with type I IFNs [25]. A notable exception to this rule are the plasmocytoid dendritic cells that have high constitutive expression levels of IRF7 [10,26,27]. TLR3 uses the adapter protein TRIF and the kinase TBK1 to mainly activate IRF3 in conventional DCs and macrophages, whereas TLR7 and TLR9 induce the expression and secretion of large amounts of type I IFNs in plasmacytoid DCs through the adaptor molecule MyD88 that directly interacts with IRF7 (not IRF3) [28,29]. The MyD88 pathway requires the IRAK4-IRAK1-IKKα kinase cascade to activate both IRF7 and the NF-κB pathway [30].

The cytosolic pathway of type I IFN induction is initiated by the recognition of viral 5′triphosphate RNA and dsRNA by RIG-I and MDA5. Binding of viral RNA leads to a conformational change of these sensors that results in their binding to MAVS (also called IPS-1, Cardif, VISA), an essential downstream adaptor in the cytosolic pathway [31–34]. Through as yet unidentified mediators, MAVS propagates the signal to the TBK1 and IKKi kinases that finally activate IRF3 and NF-κB (Figure 1).

Activated IRF3, NF-κB and ATF2/c-jun bind to the IFN-β gene promoter and induce gene transcription. IFN-β is secreted by the cells, and binds to the IFNAR in an autocrine or paracrine way. The activation of the Jak-STAT pathway upon IFNAR binding by IFN-β induces an important positive feedback loop that allows a very rapid and strong induction of an antiviral state in the infected cells and their neighbors (Figure 1).

## 4. HCV Interference with Cellular Sensors

The infection of cells with HCV leads to the induction of IFN-β through activation of the RIG-I and TLR3 pathways [19,35,36]. Interestingly, the HCV NS3/4A protease has been shown to cleave and inactivate MAVS (Cardif, IPS-1, VISA) and TRIF, two important adaptor proteins in the RIG-I and the TLR3 pathway, respectively [32,35]. The inhibition of these cellular sensors could be one of the factors contributing to viral persistence. However, as outlined in the next paragraph, HCV infection actually induces the endogenous IFN system despite HCV’s ability to cleave TRIF and MAVS.

## 5. Induction of Interferon Stimulated Genes by HCV

Hepatic gene expression studies with chimpanzees and humans provide compelling evidence that the inhibition of IFN-β induction, by cleavage of MAVS and/or TRIF, is in many cases incomplete and cannot prevent the activation of the endogenous IFN system in the liver. Acute infection of chimpanzees with HCV leads to the rapid activation of the endogenous IFN system [37]. Moreover, chronically HCV infected chimpanzees have an ongoing induction of a large number of IFN-stimulated genes (ISGs) in the liver, suggesting a continuous stimulation of the endogenous IFN system [38]. Interestingly, a single chimpanzee infected with a genotype (GT) 3 HCV showed less induction of ISGs compared to genotype 1 infected animals [38]. Many patients with chronic hepatitis C (CHC) also have a permanent induction of ISGs in the liver [39–41]. There is a strong association between such pre-activation of the endogenous IFN system and the failure to respond to pegIFNα/ribavirin therapy [39–41]. Interestingly, patients with a pre-activated IFN system have ISG expression levels comparable to those achieved in responders by the treatment with pegIFNα, and it is presently not known, why such a high expression level of ISGs in pre-activated patients does not induce a spontaneous clearance of HCV [40]. Patients with GT 1 infections significantly more often had a pre-activated IFN system than those infected with GTs 2 and 3, providing a possible explanation why the treatment is more often successful in the latter group [40].

## 6. Interferon signaling through the Jak-STAT pathway

### 6.1. The receptor-kinase complex

All type I IFNs bind to the same interferon alpha/beta receptor (IFNAR) that consists of two major subunits: IFNAR1 (the a subunit in the older literature) [4] and IFNAR2c (the βL subunit) [5,6]. Each receptor subunit constitutively binds to a single specific member of the Janus kinase (Jak) family: IFNAR1 to tyrosine kinase 2 (TYK2) and IFNAR2c to JAK1. When type I IFNs bind to the two receptor chains, TYK2 and JAK1 transactivate each other by mutual tyrosine phosphorylation, and then initiate a cascade of tyrosine phosphorylation events on the intracellular domains of the receptors and on signal transducer and activator of transcription (STAT) 1, STAT2 and STAT3.

### 6.2. Signal Transducers and Activators of Transcription (STATs)

**Figure 2.**
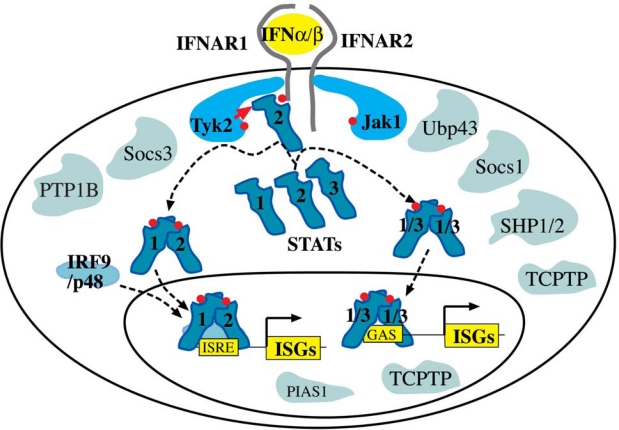
Type I IFNs signal through the Jak-STAT pathway. PTP1B, protein tyrosine phosphatase 1B; SHP1/2, SH2 domain-containing protein tyrosine phosphatase1 or 2; TCPTP, T cell protein tyrosine phosphatase.

In most cells, type I IFNs activate STAT1, STAT2 and STAT3. STAT1 and STAT2 combine with a third transcription factor, IRF9, to form interferon stimulated gene factor 3 (ISGF3). ISGF3 binds to interferon stimulated response elements (ISREs) in the promoters of ISGs. Alternatively, IFN activated STAT1 and STAT3 can form homodimers or STAT1-STAT3 heterodimers. These STAT dimers bind a different class of response elements, the gamma activated sequence (GAS) elements. Once bound to the promoters of ISGs, STATs induce the transcription of genes involved in the generation of an antiviral state (Figure 2) [42,43].

STAT proteins are composed of 750 to 850 amino acids. They share well-defined, structurally and functionally conserved domains including the amino-terminal (NH2), coiled-coil, DNA-binding, linker, SH2, tyrosine activation, and transcriptional activation domains [44].

### 6.3. Negative Regulators of Interferon Signaling

#### 6.3.1. Suppressor of Cytokine Signaling (SOCS)

SOCS proteins are important negative regulators of Jak-STAT signaling [45]. The family consists of eight members, CIS and SOCS1 to SOCS7. CIS, SOCS1, SOCS2 and SOCS3 are induced by a large number of cytokines and inhibit cytokine receptors in a negative feedback loop. Type I IFNs induce SOCS1 and SOCS3 [46], and overexpression experiments have demonstrated that both inhibit IFN signaling through the Jak-STAT pathway [46,47]. SOCS1-deficient mice develop severe inflammatory disease because of IFN-γ hypersensitivity [48], but are very resistant to viral infections, most likely because of enhanced type I IFN signaling [49].

#### 6.3.2. USP18

Ubiquitin specific peptidase 18 (USP18/UBP43) is another important negative regulator in type I IFN signaling. USP18/UBP43 was originally identified as a protease cleaving ubiquitin-like modifier ISG15 from target proteins, but was recently found to play a negative regulatory role independently of its ISG-deconjugating ability [50,51]. UBP43 was reported to inhibit the activation of JAK1 by interfering with the binding of JAK1 to IFNAR2c [52]. UBP43 deficient mice show a severe phenotype characterized by brain cell injury, poly-I:C hypersensitivity, and premature death [53,54]. Interestingly, they are resistant to otherwise fatal cerebral infections with LCMV and VSV [55]. USP18/UBP43 is also important for long-term refractoriness of the IFN system [56].

#### 6.3.3. Protein Inhibitor of Activated STAT1 (PIAS1) and PIAS3

PIAS1 and PIAS3 specifically bind to tyrosine phosphorylated STAT1 and STAT3, respectively, and inhibit the DNA-binding of STAT dimers [57]. PIAS1 selectively inhibits interferon-inducible genes and is important in innate immunity. As a consequence, PIAS1 deficient mice show increased protection against pathogenic infection [58].

#### 6.3.4. TcPTP

STAT1 is deactivated in the nucleaus by dephosphorylation of the tyrosine 701 by T cell protein tyrosine phosphatase (TcPTP) [59]. TcPTP deficient mice develop progressive systemic inflammatory disease as shown by chronic myocarditis, gastritis, nephritis, and sialadenitis as well as elevated serum IFN-γ [60].

## 7. Interference of HCV with IFN Signaling through the Jak-STAT pathway

In order to escape from the powerful antiviral effects of the IFN system, many viruses have evolved strategies to block IFN signal transduction [61,62]. Interference of HCV with IFN signaling has been suggested in several studies, sometimes with controversial results [63,64]. One proposed mechanism is the inhibition of STAT1 activation through an upregulation of SOCS3 by HCV core protein, which has been found after transient transfection of HepG2 and Huh7 cells [65,66]. Another group reported that the expression of HCV proteins in Huh7 cells leads to a proteasome-dependent degradation of STAT1 [67]. A third group, also using HCV protein expression in Huh7 cells, reported normal STAT1 expression and phosphorylation, but an inhibition of nuclear translocation of phosphorylated STAT1 [68]. A fourth group reported found reduced STAT3 expression levels in livers of patients with chronic hepatitis C and reported that HCV expression in Huh7 cells inhibited IFN-α induced phosphorylation of STAT1, STAT2 and STAT3 [69]. We have found an inhibition of DNA binding of activated STATs not only in cells transfected with the HCV genome, but also in the liver of HCV transgenic mice, and in liver biopsies of patients with CHC [70,71]. In all cases, STAT1 protein expression and tyrosine phosphorylation were not impaired.

**Figure 3.**
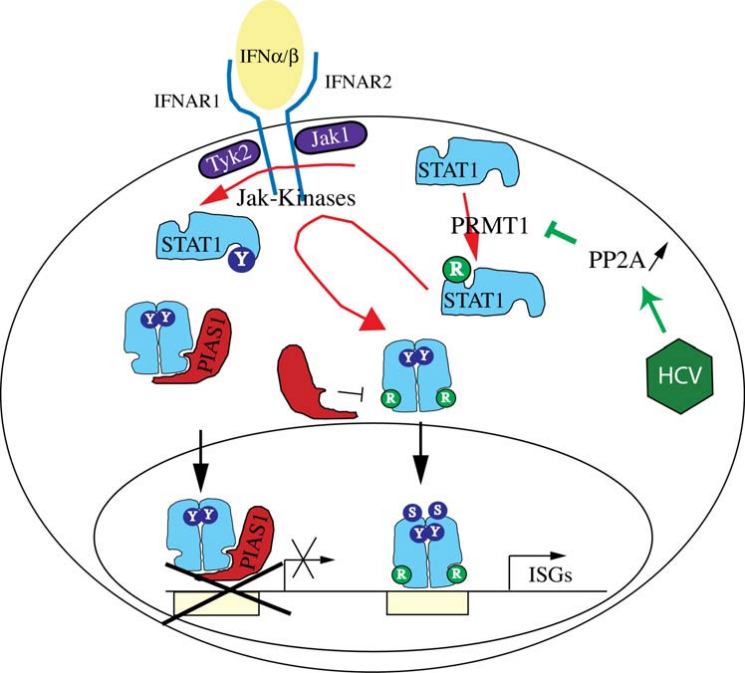
HCV induces the over-expression of PP2Ac via an ER stress response pathway. PP2A inhibits PRMT1, the enzyme responsible for STAT1 methylation. The resulting hypomethylation of STAT1 facilitates the binding of PIAS1, an inhibitor of DNA binding of activated STAT1. Tyrosine phosphorylation is shown by Y, and arginine methylation by R.

Further investigations of the molecular mechanisms of HCV interference with IFN signaling identified protein phosphatase 2A (PP2A) as an important mediator in the inhibitory pathway [72]. The catalytic subunit of PP2A, PP2Ac, was found to be overexpressed as a result of an endoplasmatic reticulum (ER) stress response induced by HCV protein expression [73]. PP2Ac was overexpressed in cells after HCV protein expression, in liver extracts of HCV transgenic mice, and in liver biopsies of patients with CHC [72]. Furthermore, expression of a constitutive active form of PP2Ac in Huh7 cells resulted in an inhibition of STAT1 DNA binding [72]. PP2A can directly bind to protein arginine methyltransferase 1 (PRMT1) and inhibit its enzymatic activity [74]. This inhibition of PRMT1 results in a decreased methylation of a number of proteins, amongst them STAT1 [72]. It has been reported that the arginine methylation of STAT1 regulates the association of STAT1 with the inhibitor PIAS1 [75], a finding that is still controversial [76]. Nonetheless, we have found that inhibition of PRMT1 by increased expression of PP2Ac leads to an increased association of STAT1 with PIAS1, a finding that could well explain the impaired DNA binding of activated STATs in HCV infected cells [72]. Interestingly, treatment of cells with the methyl group donor S-adenosyl-methionine restored normal IFN signaling in cells with HCV protein expression and increased the potency of IFNα in the HCV replicon system [77]. Our current working model of HCV interference with IFN signaling is shown in Figure 3.

## 8. Effects of Type I Interferons

Interferons exhibit a wide spectrum of biological activities in target cells, including antiviral, immunomodulatory, antiangiogenic, and growth inhibitory effects. They exert their effects mainly through Jak-STAT mediated regulation of gene transcription. However, there are also Jak-STAT independent effects, notably the activation of the p38 Map kinase signaling cascade [78,79], and the activation of the phosphatidylinositol 3 (PI3) kinase – Akt kinase – mTOR/p70 S6 kinase pathway that regulates mRNA translation [80,81].

### 8.1. Interferon Regulated Genes

Stimulation of cells with type I IFNs usually leads to the induction of several hundred genes (IFN stimulated genes, ISGs), but there are also some genes that are negatively regulated by IFNs [40,82,83]. There is considerable variation between different cell types in regard to the number and also the identity of the regulated genes [83]. Gene expression analysis in human and chimpanzee have shown that systemic administration of (pegylated) IFNα induces overlapping but clearly distinct sets of genes in liver and peripheral blood mononuclear cells (PBMCs) [40,84]. The mRNA levels of most of the genes are increased 2 to 10 fold by IFN stimulation, but some genes are induced even stronger [40]. In the liver, most of the ISGs are upregulated within hours after administration of pegylated IFNα and rapidly downregulated again within the first 8 to 24 hours [84].

### 8.2. Antiviral Effects

Type I IFN induced regulation of hundreds of genes establishes an “antiviral state” in the cell [85,86]. The term “antiviral state” implies protection of the cell against viral infection, but it is a generic term, and the lack of precise criteria for its definition reflects the fact that we still have only an elementary understanding of what exactly it is. Indeed, a large number of these regulated genes have as yet unknown functions. Some ISGs have broad antiviral effects. For example, protein kinase R (PKR), a member of the eukaryotic initiation factor 2α (eIF2α) kinase family, phosphorylates eIF2α with a consequent blockade of translation of most cellular and viral mRNAs [87]. Members of the interferon-induced protein with tetratricopeptide repeats (IFIT1 (ISG56) and IFIT2 (ISG54)) also inhibit translation by binding to eIF3 [88]. Another well-studied antiviral effector is 2′–5′ oligoadenylate synthetase (OAS). Both the gene transcription and the enzymatic activity are regulated: the enzymatic activity is stimulated by viral dsRNA, and OAS expression is upregulated several-fold by IFN-α. The 2′–5′oligoadenylates produced by activated OAS in turn activate the latent RNA nuclease RNase L, resulting in the degradation of viral and host RNAs [87]. Recently, the ISG15 system has been found to be another broadly active non-specific antiviral effector. ISG15 is one of the most prominent ISGs. It is an ubiquitin-like protein that conjugates to more than 150 cellular target proteins [55,89–91]. The conjugation is executed by an enzymatic cascade that includes an E1 activating enzyme (UBE1L) [92], an E2 conjugating enzyme (UbcH8) [93,94], and an E3 ligase (HERC5 and TRIM25) [95,96]. The conjugation can be reversed by ubiquitin protease 43 (UBP43, also known as USP18) [51]. All these enzymes are induced by type I IFNs. Many of the ISG15 target proteins have important roles in the IFN response, for example Jak1, STAT1, RIG-I, MxA, PKR and RNaseL [90]. Consistent with its role in the IFN system, mice deficient in ISG15 have increased susceptibility to infection with several viruses [97].

Several ISGs have been implicated in the host defence against hepatitis C virus (HCV). Viperin, a member of the radical S-adenosyl methionine domain containing enzymes, inhibits replication of HCV in the replicon system [98,99]. PKR and ISG20, a 3′–5′ exonuclease with a strong preference for single-stranded RNA, also strongly inhibit HCV replicons [99].

## 9. Interference of Hepatitis C Virus with Interferon Effector Systems

The HCV protein NS5A was shown to inhibit PKR activation in cell culture [100]. In some populations, mutations in the so-called IFN sensitivity-determining region (ISDR) of HCV NS5A correlate with response to IFN treatments, and the ISDR is part of the PKR binding domain [101]. This finding, however, was not confirmed in other populations.

Likewise, HCV E2 was shown to bind to PKR through a 12-amino acid sequence similar to the PKR autophosphorylation site and the eIF2α phosphorylation site, the PKR-eIF2α phosphorylation homology domain (PePHD). In this elegant work using transfected cells and yeast, E2 blocked the inhibitory effect of PKR on protein synthesis and cell growth. Interestingly, the homology of this 12-amino acid sequence of E2 was found only in HCV GT 1a and 1b, but not GT 2a, 2b or 3a, providing a potential explanation for the better response rates obtained in patients with GT 2 or GT 3 infections [102]. However, a clinical study could not find a significant correlation between the presence of this 12-amino acid motif and response to IFN therapy [103].

## 10. Conclusions

HCV is exceptionally successful in establishing a persistent infection, and must have therefore evolved mechanisms to interfere with the powerful innate immune system. There is evidence mainly from cell culture work that HCV interferes with the induction of IFNβ in infected cells, with IFNα signaling trough the Jak-STAT pathway, and with IFN induced effector mechanisms, e.g. PKR. However, the significance of these mechanisms *in vivo* is less clear, and further work with liver biopsy samples obtained from patients treated within the framework of rigorously designed clinical trials will have to identify the mechanisms of viral interference that allow HCV to persist chronically in such a high percentage of infected individuals.
